# The Evolution of Fitness Landscapes: From Visualizing Evolution to Controlling It

**DOI:** 10.3390/cancers18183014

**Published:** 2026-09-17

**Authors:** Meaghan Elizabeth Parks, Peng Chen, Jinling Wu, Jacob G. Scott

**Affiliations:** 1 Genomic Sciences and Systems Biology, Cleveland Clinic Research, Cleveland, OH 44106, USA; mep171@case.edu (M.E.P.); pxc544@case.edu (P.C.); 2 School of Medicine, Case Western Reserve University, Cleveland, OH 44106, USA; 3 Department of Physics, Case Western Reserve University, Cleveland, OH 44106, USA

**Keywords:** fitness landscape, Fisher’s geometric model, evolutionary control

## Abstract

Fitness landscapes have long served as a conceptual tool in evolutionary biology. In recent years, however, they have shifted from being viewed primarily as theoretical constructs to being used to interpret experimental data and, increasingly, to guide evolutionary outcomes in biomedical systems. In this review, we trace that progression across major landscape formalisms, including Fisher’s geometric model, Wright’s adaptive landscape, fitness seascapes, and empirical fitness landscapes. We aim to highlight the strengths of each model and its potential for use in cancer research. Rather than treating these frameworks as isolated models, we show how they collectively reflect the field’s maturation: from describing adaptation in idealized settings to quantifying epistasis and evolutionary accessibility, to interpreting experimental genotype–fitness data, and finally to motivating strategies for steering evolution in contexts such as treatment resistance in cancer. We argue that the central promise of the fitness landscape framework now lies not only in explaining evolutionary dynamics, but also in enabling predictive interventions and overcoming cancer evolution.

## Simple Summary

Cancer evolves over time, allowing tumors to adapt to treatment and develop resistance. Fitness landscapes provide a way to visualize and study this evolutionary process by linking genetic or phenotypic changes to their effects on fitness. In this review, we describe how fitness landscapes have developed from theoretical models into empirical and dynamic frameworks that can incorporate experimental data and changing environments. We discuss major landscape models and how these approaches may be used to predict evolutionary outcomes and guide treatment strategies. Although the clinical application of evolutionary control remains limited, continued integration of experimental, computational, and clinical data may enable fitness landscapes to become useful tools for predicting and ultimately controlling cancer evolution.

## 1. Introduction

Our inability to predict and control cancer’s capacity to evolve resistance is the largest challenge facing modern oncology. The first step toward controlling or preventing treatment resistance is understanding how evolution occurs. The need to understand evolution has long been evident. One of the most influential conceptual models for understanding evolution is the fitness landscape, which has evolved from a simple theoretical concept into a tool for interpreting real-world biological data in recent years. In this review, we trace the development of fitness landscapes from conceptual metaphors to formal mathematical models, which then became empirical tools and modern evolutionary frameworks, highlighting their growing potential not only to describe, but also to steer evolutionary results, particularly in cancer.

Fitness landscapes were first conceived in the 1930s by Ronald Fisher and Sewall Wright. Fitness landscapes provide a conceptual framework for visualizing how genotypes or phenotypes map to fitness, defined as reproductive success [1,2]. Each point in the landscape represents a specific genetic or phenotypic state, with elevation corresponding to its associated fitness. Both Fisher and Wright independently proposed fitness landscape models; as such, these models differ substantially and inspired a wide range of more complex models to address new insights in biology [1,2]. Fisher proposed a smooth model based in phenotypic space, whereas Wright proposed a rugged model in genotypic space [1,2]. We discuss both models further later in the paper. Although influential, several landscapes have not seen major use in cancer research; therefore, we will not focus on them. Such models include Waddington’s landscape [3], which has been a useful tool and inspired many research projects outside of the biomedical research field, and Simpson’s concept of adaptive zones [4], which is much the same; as such, while important concepts and worthy of mention, they will not be the focal point of this article. Instead, this article will focus on Wright and Fisher’s models and those inspired by them, as they are the prominent models in cancer research, mainly for their flexibility, potential for extension, and demonstrated success in predicting real-world phenomena.

The distinction between rugged and smooth landscapes continued with more complex rugged landscapes, such as Kauffman’s NK model, which was heavily inspired by Wright’s work. The more rugged models gained traction in the scientific community over time as the importance of epistasis became clearer. However, that is not to say that smooth fitness landscapes, those inspired by Fisher’s model, were of no interest, and extensive work has been done to extend the original model [1,5,6,7,8] to address real-world questions.

**Figure 1:**
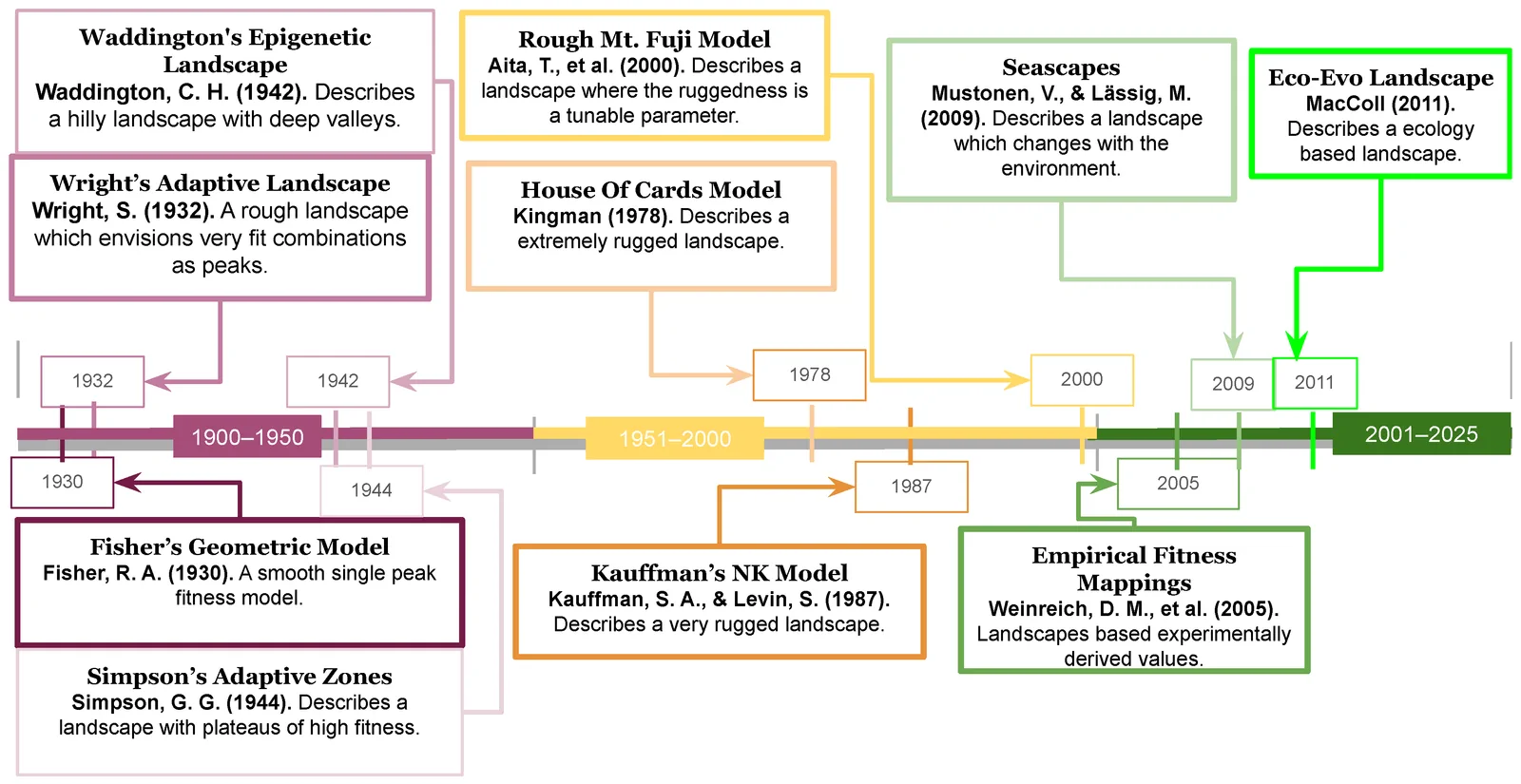
Timeline of seminal publications introducing major fitness landscape models and their authors. The works are organized into three time periods according to their publication dates, beginning with the pioneering contributions of Wright and Fisher [1,2,3,4,8,9,11,12,13,14].

Later models, such as the Rough Mt. Fuji model, were proposed, which, in some respects, bridge the divide between rough and smooth landscapes by making the landscape’s ruggedness, and therefore the levels of epistasis, a tunable parameter [9]. Another example that merges Fisher and Wright-inspired models is empirical fitness mappings, in which, instead of fitting an existing model to data, the landscape is uncovered through experimental measurements or inference rather than assuming predetermined ruggedness. Empirical landscapes may be (1) complete combinatorial, with all genotypes measured within a defined mutational space; (2) partially sampled, with only a subset of genotypes characterized; or (3) inferred, with genotype–fitness relationships reconstructed from incomplete observations. Thus, their ruggedness is an empirical property of the genotype–fitness relationship rather than a consequence of how the landscape is obtained. The final model we discuss is the fitness seascape, which changes to mimic evolving environments and thus may result in the landscape exhibiting varying levels of epistasis depending on the environment [10,11,12]. The inception of each of these models is shown in Figure 1, which indicates when some of the most influential papers for each model were published, beginning with the landmark papers by Wright and Fisher.

While much of the research on fitness landscapes is theoretical, advances in biology and technology have enabled their use, moving from conceptual models to tools directly applicable to experimental data and that can further be used clinically to guide evolution. The availability of gene editing and sequencing has produced an immense amount of genotype and phenotype data; combining this with our ability to measure growth as a proxy for fitness has led to a surge in papers using fitness landscapes to interpret these data in novel ways. However, given the wide range of applications and the variety of landscapes in evolutionary biology and oncology, it can be difficult to understand the current state of the field. This review examines fitness landscapes and argues for their broader use in cancer research. We begin with the concepts that shape landscape structure, including epistasis and evolutionary accessibility, and then review foundational theoretical models before turning to empirical fitness landscapes and seascapes. We conclude with emerging efforts to use landscape-based methods to predict and steer evolution in cancer, with the ultimate goal of achieving evolutionary control.

## 2. Epistasis, Ruggedness and Accessibility on Fitness Landscapes

**Figure 2:**
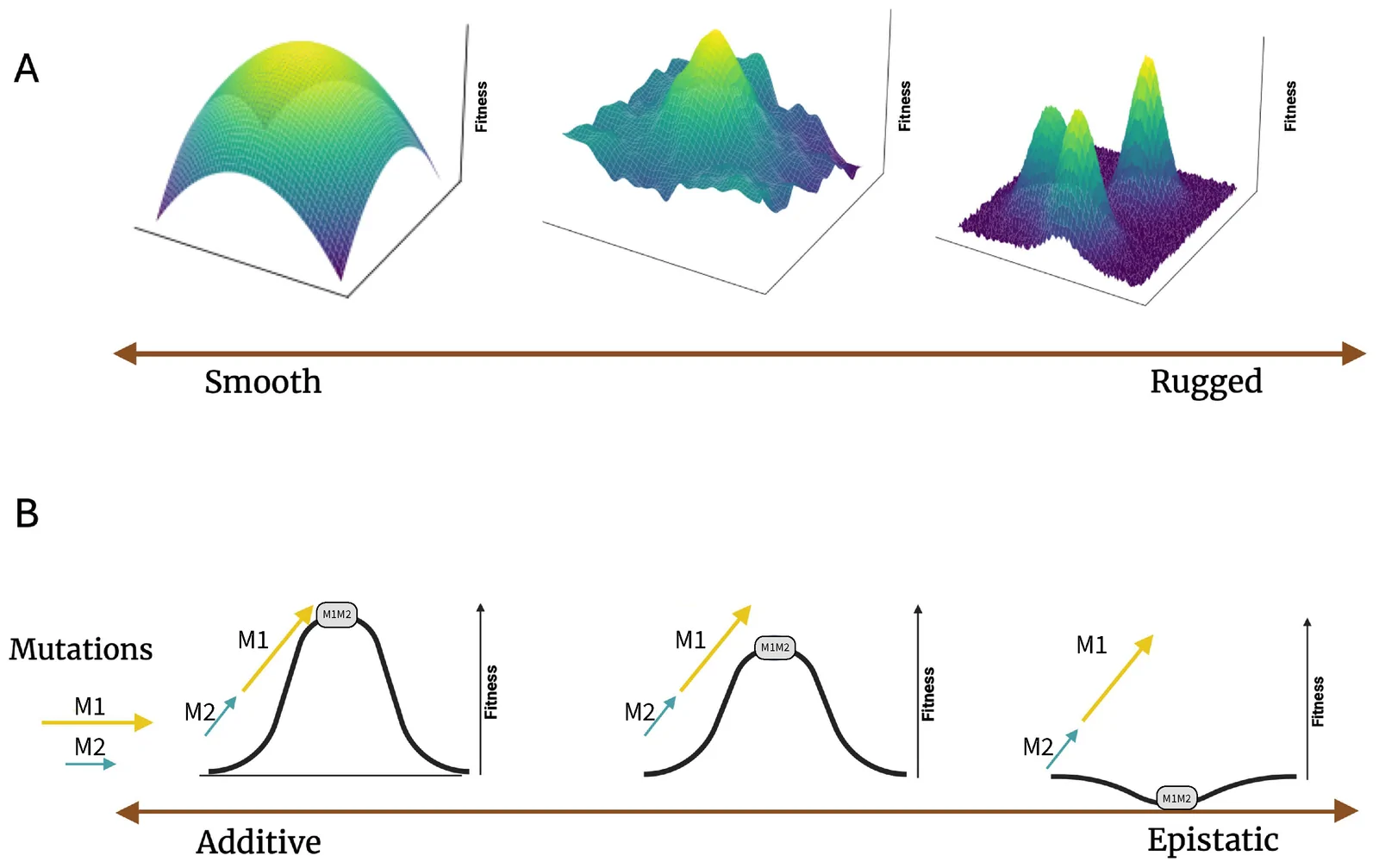
Variety of forms fitness landscapes can take and how epistasis affects behavior. (**A**) Fitness landscapes range from smooth, single-peak landscapes to increasingly rugged landscapes with multiple local optima. (**B**) A range from additive interactions, where genes contribute independently to fitness, to epistatic interactions, where genes can interact negatively within the fitness landscape.

Landscape structure is governed primarily by the pattern of genetic interactions, or epistasis, a phenomenon that describes how the fitness effect of a mutation depends on the genetic background in which it occurs [15,16,17]. Many studies have demonstrated the importance of epistasis in cancer, as the inconsistent effects of mutations make predictions about evolution challenging, making it an essential factor in studying cancer evolution and treatment resistance [18,19,20]. When contributions of individual mutations are approximately additive, and epistasis is weak or non-existent, landscapes are effectively smooth and have few peaks: fitness tends to increase along many mutational directions, and evolution can progress predictably toward a single dominant peak. In contrast, strong epistasis can generate rugged landscapes with multiple local optima separated by low-fitness valleys because mutations’ effects on fitness are inconsistent. Figure 2A illustrates these qualitative regimes, from smooth single-peak landscapes to highly rugged surfaces shaped by pervasive epistasis. When two genes are purely additive, the fitness is the sum of the fitness benefits from the genes; the landscape illustrates this on the far left of Figure 2B. In contrast, two genes can interact antagonistically, resulting in sign epistasis; in the extreme case of reciprocal sign epistasis, the fitness effects of both mutations change sign depending on the genetic background, as illustrated in the landscape farthest to the right in Figure 2B. Intermediate landscape structures can exhibit magnitude epistasis, including diminishing-returns epistasis, without necessarily producing multiple fitness peaks.

At the opposite extreme lies the House-of-Cards (HoC) model, in which fitness values are assigned independently and at random to each genotype [13,21]. HoC landscapes represent a maximally epistatic limit, characterized by extreme ruggedness, an exponential number of local fitness peaks, and minimal correlation between neighboring genotypes. As a result, adaptive paths are highly constrained, and evolutionary dynamics are dominated by local fitness comparisons rather than global structure, unlike models with weaker epistasis.

Between these two extremes lie the vast majority of biologically relevant fitness landscapes, where epistasis is neither absent nor completely random but structured. A particularly important class of interactions is sign epistasis, in which a mutation that is beneficial in one genetic background is deleterious in another. Sign epistasis restricts the order in which mutations can accumulate and removes certain mutational paths from adaptive walks [11]. An even stronger constraint arises from reciprocal sign epistasis, where the effects of two mutations each change sign depending on genetic background; this interaction is a necessary condition for the existence of multiple fitness peaks in discrete genotype spaces [22].

Beyond pairwise interactions, higher-order epistasis refers to fitness effects arising from interactions among three or more loci that cannot be decomposed into sums of lower-order terms. Such interactions can qualitatively reshape fitness landscapes, modifying peak structure, basin geometry, and evolutionary predictability even when pairwise epistasis is weak or absent [23]. Recent work has also emphasized the role of global epistasis, where systematic background-dependent effects induce nonlinear but structured fitness responses across large regions of genotype space, even when local interactions appear noisy, and is an important element when making more general predictions about a fitness landscape’s shape [24].

These epistatic features directly influence accessibility, how easily an organism can reach the landscape’s peak, defined as the existence of mutational paths that monotonically increase fitness between genotypes. In smooth landscapes, accessibility is typically high, while in HoC-like or strongly rugged landscapes, most high-fitness genotypes are evolutionarily inaccessible under strong selection because all connecting paths involve transient fitness decreases [25]. Importantly, selection itself can bias the prevalence and type of epistatic interactions encountered along adaptive trajectories, shaping both local accessibility and global landscape structure [26,27].

Evolution on fitness landscapes can be pictured as movement driven by mutation and biased by selection toward higher-fitness regions. On smooth landscapes, this motion resembles a directed climb, whereas on rugged landscapes, populations may become trapped at local peaks and occasionally cross low-fitness valleys to reach higher peaks. Depending on population-genetic parameters, evolutionary trajectories can resemble biased random walks on the genotype graph, with outcomes determined by both landscape topography and stochastic sampling. A particularly well-studied regime is strong selection, weak mutation (SSWM) [21], in which beneficial mutations arise infrequently enough that the population is effectively monomorphic, consisting mainly of a single genotype or phenotype, between fixation events. The population can then be represented as a single point on the landscape that hops between neighboring genotypes by rare substitutions, producing an “adaptive walk” on a genotype or phenotype graph. The SSWM approximation underlies many theoretical results connecting landscape features (e.g., peak structure, epistasis, and accessibility) to predictability and rates of adaptation, and it provides an intuitive bridge between population-genetic dynamics and geometric landscape representations. Because of the wide range of epistatic interactions, the many ways it can influence disease dynamics, and their known importance in cancer, having different landscape models that capture distinct aspects and levels of epistasis is valuable for efforts to understand and ultimately control cancer evolution.

## 3. Fisher’s Geometric Model (1930)

Fisher’s Geometric Model (FGM) was published in 1930 and is a smooth single-peak model in which the axes represent independent phenotypic traits that contribute to fitness [1]. An organism is represented by a phenotypic vector z

(1) $$z=(z_{1},\ldots ,z_{D})$$

where each component $z_{i}$ represents a quantitative phenotypic trait, and *D* is the dimensionality of the phenotypic space. Genetic mutations act upstream of this representation by altering one or more phenotypic traits, thereby moving the organism through phenotypic space. A mutation can therefore be represented as a displacement Δz such that the resulting phenotype is $z^{\prime }=z+\Delta z$. Different mutations may have different directions and magnitudes in phenotypic space, while a given mutation is typically assumed to have a consistent phenotypic effect across genetic backgrounds in the canonical formulation of FGM.

The relative fitness (*w*) of an organism is defined as the distance from the optimal combination (*o*) of traits at the peak, with *k* determining the rate of decline in fitness with distance from the peak, or in other words, the width of the peak. 

(2) $$w\left(z\right)=\exp\left[-{\left(\sum_{i=1}^{D}{\left(z_{i}-o_{i}\right)}^{2}\right)}^{k/2}\right],$$

The assumptions within the model are: one, that there is a single optimal phenotypic combination, and two, that each individual mutation will have consistent effects and move an organism in the same direction and by the same magnitude, so organisms close to the peak will almost always suffer from large-effect mutations [1]. As such, FGM is a particularly effective representation of sign epistasis, where, depending on the context, a mutation can have a positive or negative effect on fitness. The inclusion of sign epistasis makes it a good option for cancer research, where epistasis and particularly sign epistasis are widely observed [18,19].

### Fisher’s Geometric Model Extensions

FGM has evolved through contributions from other researchers, with plenty of room for further growth, particularly in cancer research. This section briefly summarizes the most important extensions of the model, which allow for the transition from a theoretical tool to one used to explain phenomena in experimental data and guide cancer. Two researchers provided the majority of the extensions to the model and conducted extensive theoretical work to elucidate its behavior, both allowing the model to be applied to a wider number of questions and increasing confidence in the model’s ability to represent real-life phenomena [5,6,7,28,29]. These extensions included extending the model to study how the number of independent phenotypic traits affects its dynamics and creating a framework to examine how multiple traits interact, thereby increasing the model’s potential research applications [5,6,7,28,29]. Additionally, these same researchers demonstrated how adaptive walks can be studied in the landscape and showed the distribution of fitness effects and that the model can represent complex systems [5,6,7,28,29].

This extensive theoretical work and detailed model characterization have also allowed researchers to apply the model to experimental data. This work generally focuses on the distribution of fitness effects, speciation, and epistasis, all important topics in biomedical research, with the distribution of fitness effects and epistasis being relevant in cancer research [30,31,32,33,34,35]. These studies using experimental data have shown that FGM has great potential as a model of evolution in real-world contexts. However, applications in medicine have been extremely limited, with most work focusing on bacterial evolution [34,36]. Recently, Razo-Mejia et al. developed a method using autoencoders to apply FGM to fitness measurements of *Escherichia coli* under treatment stress; their work demonstrated that FGM can be used to analyze real-world data and potentially predict evolutionary outcomes [36].

The model’s success in these instances is extremely promising for future applications, and its extensions make it an ideal option for addressing a range of cancer-related challenges. Examples include applying a method similar to that of Harmand et al. or Razo-Mejia et al. to study cancer evolution under different treatments, or using Orr’s method to perform adaptive walks with available genetic knockout data to predict how a tumor may evolve [5,6,7,34].

## 4. Wright’s Adaptive Landscape (1932)

Sewall Wright’s concept [2] of the adaptive landscape remains one of the most influential metaphors in evolutionary biology, providing a topographic framework for understanding how genetic variation, selection, mutation, and drift jointly shape evolutionary dynamics. First articulated in Wright’s foundational work on Mendelian populations, the adaptive landscape maps genotype to fitness, with evolutionary change visualized as population movement across a multidimensional surface defined by peaks, valleys, and ridges of fitness [2,37].

In this representation, each genotype occupies a node in genotype space, and mutational neighbors are connected by edges corresponding to single mutational steps. For biallelic loci, this structure naturally forms an *L*-dimensional hypercube with $2^{L}$ genotypes, where evolutionary dynamics unfold as movement along edges driven by mutation and biased by selection. Fitness differences between neighboring genotypes define local slopes on the landscape, guiding adaptive change under selection. A central insight of Wright’s adaptive landscape is that adaptation need not be globally optimizing. In contrast to the smooth, single-peak landscapes implicit in simple phenotypic models, genotypic landscapes may contain multiple local fitness peaks, separated by valleys of reduced fitness. Under SSWM-like dynamics, populations evolving under selection can therefore become trapped on suboptimal peaks, with further adaptation requiring stochastic effects such as genetic drift, population subdivision, or environmental change to enable transitions between peaks [2].

Although originally introduced as a heuristic metaphor, the adaptive landscape has since been formalized in a wide range of mathematical and computational models. Two canonical landscape models, the NK model [8] and the Rough Mount Fuji (RMF) model [38], play a dual role in contemporary research. First, they serve as generative tools: by tuning parameters that control the structure and degree of landscape ruggedness, researchers can produce ensembles of synthetic landscapes to study, in silico, how landscape topography shapes evolutionary dynamics, adaptive walk statistics, and the prevalence of local optima. Second, these models provide interpretive benchmarks for empirical landscapes: experimentally measured genotype–fitness maps can be compared with NK- or RMF-generated ensembles, and model parameters can in some cases be estimated from empirical data, allowing researchers to characterize landscape ruggedness, epistatic structure, and evolutionary accessibility [39,40]. This dual usage, synthetic generation, and empirical interpretation have made these models widely used in evolutionary biology and, increasingly, in biomedical research on antimicrobial resistance [41] and, more relevantly, cancer progression [42,43].

### 4.1. Canonical Models

#### 4.1.1. Kauffman’s NK Model (1987)

The NK model, introduced by Kauffman et al. [8,44], provides a mathematically tractable framework for constructing genotype–fitness landscapes with tunable ruggedness. In this model, a genotype is represented as a binary string of length N,

(3) $$g=\left(g_{1},\ldots ,g_{N}\right),\;g_{i}\in \left\{0,1\right\},$$

embedded in a Hamming graph where edges correspond to single mutations. Fitness is defined as an aggregate of locus-specific contributions,

(4) $$F\left(g\right)=\frac{1}{N}\sum_{i=1}^{N}f_{i}\left(g_{i};g_{i1},\ldots ,g_{iK}\right),$$

where each contribution $f_{i}$ depends on the state of locus *i* and on the state of *K* other loci specified by a prescribed interaction structure. Each $f_{i}$ is sampled independently from a fixed distribution, yielding a quenched random landscape. The parameter *K* controls the degree of epistasis by specifying how many additional loci influence the fitness contribution of each site. When K=0, the landscape is purely additive and smooth, possessing a single global optimum. In contrast, K=N−1 yields the uncorrelated HoC limit with maximal ruggedness. Intermediate values of *K* interpolate between these extremes and generally increasing the prevalence of sign epistasis, the number of adaptive peaks, and overall landscape ruggedness.

When applying the NK model in a cancer context, *K* can be interpreted as an effective measure of epistatic connectivity among the genetic alterations represented in the model. If the *N* loci represent oncogenic or tumor-suppressor alterations, K=0 corresponds to a model in which their fitness contributions are independent, whereas increasing *K* increases epistatic connectivity and the potential for context-dependent fitness effects among these alterations. Such context dependence has been observed experimentally in cancer, where the fitness effects of tumor-suppressor alterations can vary substantially across oncogenic backgrounds [45]. Importantly, *K* should not be interpreted as the literal number of molecular interaction partners of a gene, but rather as a coarse-grained representation of epistatic connectivity at the scale of the model. When prior biological information is available, the interaction structure may be informed by known genetic or signaling interactions; alternatively, candidate values of *K* can be compared based on their ability to reproduce observed genotype–fitness relationships.

There has been much work to characterize the local statistical structure of NK landscapes, showing that for fixed *K*, the expected number of local optima grows exponentially with *N*, while the mean adaptive walk length decreases rapidly as *K* increases [46]. However, these properties are not determined by *K* alone, and several other factors can strongly constrain evolutionary trajectories [46,47,48]. The NK model has also been widely used to study adaptive dynamics. Although it defines a fixed genotype–fitness map, selection biases which mutations substitute along adaptive trajectories and, consequently, the prevalence and type of epistasis observed among successive substitutions. Epistasis is less prevalent than expected early in adaptation and becomes enriched later, accompanied by a shift from predominantly antagonistic interactions toward synergistic and sign epistasis [27]. Beyond its role as a theoretical tool, the NK model has been applied directly to biomedical problems. Kauffman et al. used it to model affinity maturation in the immune system, showing that tuning *K* to match the observed number of somatic hypermutation steps (∼6–8) during antibody maturation predicts features of antibody space consistent with experiment [44].

#### 4.1.2. Rough Mount Fuji Model (2000)

While NK landscapes provide a framework for modeling tunable ruggedness through explicit epistatic interactions, they offer limited analytical tractability and obscure the relative contributions of global fitness gradients and local ruggedness. The Rough Mount Fuji (RMF) fitness landscape was introduced to address these limitations by superimposing a smooth additive fitness slope onto random fitness fluctuations, thereby interpolating between a purely additive “Mount Fuji” landscape and a maximally rugged random landscape [9]. In its standard formulation, the fitness F(g) of a genotype g is given by

(5) $$F\left(g\right)=-cd\left(g,g^{*}\right)+\eta \left(g\right),$$

where $d\left(g,g^{*}\right)$ is the Hamming distance from a reference genotype $g^{*}$, which represents the fitness optimum of the additive component of the landscape; c≥0 sets the strength of the global fitness gradient; and η(g) is a random variable drawn independently for each genotype, often from a Gaussian or uniform distribution with variance $\sigma^{2}$. The ratio σ/c controls the effective ruggedness of the landscape: in the limit σ→0, the landscape is purely additive, while for c→0, the RMF reduces to a fully uncorrelated random landscape.

For empirical applications, *c* and σ can in principle be estimated from genotype–fitness measurements, with *c* capturing the global fitness trend with Hamming distance from a specified reference genotype $g^{*}$ and σ capturing deviations from this trend [9]. Reliable estimation of σ/c, however, requires adequate genotype-space coverage and measurement precision, as sparse sampling can make the two components difficult to distinguish and experimental error can inflate inferred ruggedness [49]. Replicate measurements or explicit modeling of measurement uncertainty can help reduce this bias.

Early work used the RMF framework to study adaptive walks under SSWM dynamics, showing that even weak random components can generate local fitness maxima and substantially alter evolutionary trajectories relative to purely additive landscapes [38]. Subsequent studies demonstrated that RMF landscapes capture key empirical features of protein fitness landscapes, notably the coexistence of a global fitness gradient with pronounced local ruggedness [9]. Building on this foundation, Neidhart et al. [50] placed the RMF model within a broader class of tunably rugged fitness landscapes, characterizing their topographical structure and deriving analytical results for quantities such as the expected number of local optima, the location of the global peak, the fitness correlation function, and adaptive walk statistics. A central advantage of RMF over NK landscapes is that ruggedness can be continuously varied via the noise amplitude σ without altering the dimensionality or the explicit epistatic structure of genotype space, enabling a clean separation between global fitness gradients and stochastic ruggedness.

More recently, Diaz-Uriarte used simulated RMF landscapes to study tumor evolution and evaluate cancer progression models, demonstrating that landscape ruggedness, particularly reciprocal sign epistasis, can strongly reduce the reliability of inferred mutational orderings and limit the predictability of tumor evolution [42,43]. RMF-type constructions have also been extended to alternating and correlated environments, where multiple RMF landscapes share controlled correlations between their random components η [51]. This formulation naturally connects RMF landscapes to studies of evolution under environmental switching and adaptive control, providing a framework for investigating how treatment sequencing may alter evolutionary trajectories [41,52,53].

## 5. Fitness Seascape (1994)

Classical fitness landscape theory conceptualizes evolution as an adaptive walk on a static mapping from genotype to fitness. While this framework has been instrumental in elucidating the roles of epistasis, ruggedness, and adaptive constraints, it is increasingly clear that many biological systems evolve in environments that vary over time and space. This is particularly relevant when studying diseases under treatment. David Merrell first introduced the notion of a fitness seascape, extending the landscape metaphor to account for environmentally contingent fitness effects [54]. Building on this idea, Ville Mustonen and Michael Lässig developed a formal framework for fitness seascapes that treats adaptation in fluctuating environments as an intrinsically non-equilibrium process [12]. In this formulation, selection coefficients vary in time, and adaptation reflects a competition between the pace of environmental change and the population’s capacity to respond through mutation and selection. Evolutionary outcomes therefore depend not only on the structure of the underlying landscape, but also on the temporal structure of environmental fluctuations, including their rates, amplitudes, and correlations [12,55].

Formally, a fitness seascape can be described by a time- or environment-dependent fitness function F(g)=f(g,e(t)), where *g* denotes genotype and e(t) the environmental state [12,56]. Selection is thus non-stationary, and evolutionary dynamics generally operate far from equilibrium. When environmental change is slow relative to adaptation, populations may track moving fitness optima; when it is rapid, populations may lag, exhibit transient adaptation, or face extinction [57,58].

Fitness seascapes arise naturally in biomedical contexts where environmental variables such as drug concentration vary across time and space. Pharmacokinetic processes generate time-dependent concentration profiles through absorption, distribution, metabolism, and clearance, while spatial heterogeneity can arise from drug diffusion, tissue structure, and vascular transport, producing gradients in selective pressure across infection sites or tumor regions. Together, these processes create environments in which genotype fitness depends on local drug concentration and therefore varies across both time and space [59,60]. In studies of antimicrobial resistance and cancer therapy, this dependence is often modeled explicitly using parametric response functions, commonly Hill-type dose–response relations, that map drug concentration to genotype-specific fitness [56,60,61,62]. Such formulations allow experimentally measured drug-response curves to be incorporated directly into evolutionary models of treatment response. More broadly, fitness seascapes arise naturally in systems governed by adaptational trade-offs, fluctuating stress, and therapeutic interventions, providing a unifying framework for understanding evolution in realistic, dynamically driven biological environments, such as a tumor undergoing treatment [56,60,61,63,64].

## 6. Empirical Landscapes (2005) and Their Applications

The theoretical frameworks reviewed above provide powerful tools for understanding evolutionary dynamics. However, their ultimate value lies in how effectively they can be linked to real biological systems, where fitness is estimated from experimental data rather than specified a priori by model assumptions. Such connections are particularly important in medicine, where evolutionary dynamics shape clinically relevant phenomena such as antibiotic resistance, viral escape, and tumor progression.

Early efforts to connect fitness landscape theory with empirical data emerged in systems where genotype–phenotype relationships could be experimentally manipulated and fitness reliably quantified. Beginning in the mid-2000s, advances in site-directed mutagenesis, high-throughput growth assays, and later deep mutational scanning enabled systematic measurement or inference of fitness effects across defined sets of mutations, often using experimentally tractable fitness proxies, particularly in microbial [61,65,66,67,68,69,70,71,72], protein [39,73,74,75], viral [76,77,78,79,80], and importantly cancer systems [45,81,82,83] (Table 1). Many influential empirical fitness landscapes have been constructed in the context of antibiotic or drug resistance, where fitness can be approximated by growth rate or competitive advantage under drug exposure. These systems provided an especially attractive testing ground for evolutionary theory because resistance evolution can often be measured directly in laboratory populations, underscoring the method’s potential for use in cancer research, where we have many patient-derived cell lines that could be studied.

Across the studies summarized in Table 1, several common features emerge despite differences in biological scale and fitness measurement. Epistasis and context dependence recur across microbial, protein, viral, and cancer systems, constraining accessible evolutionary trajectories and allowing environmental changes to reshape landscape topology [61,66,67,72]. Microbial and protein systems have enabled particularly dense or combinatorial sampling of genotype space, whereas cancer landscapes remain more difficult to resolve experimentally because fitness is often quantified through tumor growth or inferred from longitudinal clonal dynamics.

Nevertheless, recent cancer studies reveal substantial diversity in landscape structure. Yousefi et al. identified a smooth, fully accessible landscape of tumor suppressor alterations across multiple genetic backgrounds in lung adenocarcinoma [83], whereas Blair et al. showed that oncogenic context can markedly alter the fitness effects of tumor suppressor loss in lung adenocarcinoma [45]. Longitudinal single-cell analysis in breast cancer further demonstrated that cisplatin treatment can reorder clone-specific fitness and invert clonal fitness landscapes [82]. Together, these studies demonstrate that cancer fitness structure can be measured or inferred at both genotype and clonal levels. Related work using large cancer-sequencing cohorts has estimated mutation-specific selective advantages [84], while Cross et al. used multi-region sequencing and phylogenetic analysis to characterize the evolutionary fitness landscape of colorectal tumorigenesis [85]. Collectively, these findings indicate that resistance and clonal evolution cannot be inferred from mutation identity alone, but depend on genetic background, treatment context, and how fitness is operationalized.

Empirical fitness landscapes provide not only a framework for interpreting past evolutionary outcomes but also a basis for prospective evolutionary prediction. When genotype–fitness relationships are measured across combinatorial mutant panels, they can be used to estimate which adaptive paths are accessible, which resistance mutations are most likely to arise, and how evolution under one drug may influence sensitivity to others, a phenomenon known as collateral sensitivity. However, because evolutionary trajectories are inherently stochastic, collateral sensitivity relationships are not fixed and can vary across replicate evolutionary realizations [86,87,88]. To capture this variability, Nichol et al. introduced the concept of *collateral sensitivity likelihoods*, which quantify the probability that selection under one drug will increase sensitivity to another across replicate evolutionary outcomes [89]. These insights have motivated the development of evolutionary-informed therapeutic strategies that exploit the structure of the fitness landscape to slow or reverse resistance evolution.

Prediction can also be strengthened when the measured genotype–fitness relationship is incomplete or high-dimensional. For example, Razo-Mejia et al. [36] used geometry-informed variational autoencoders to recover latent structure in phenotype-to-fitness maps and improve prediction of out-of-sample antibiotic responses from empirical fitness measurements. Although developed in a microbial system, such inference methods illustrate how empirical landscapes can progress from retrospective descriptions of adaptation toward models capable of forecasting evolutionary responses. Collectively, these developments establish the predictive foundation required for the next step: using knowledge of the landscape not merely to anticipate evolution, but to influence its course.

### 6.1. Evolutionary Control Using Empirical Fitness Landscapes and Seascapes

Moving from prediction to steering requires using knowledge of likely evolutionary trajectories to select interventions that bias populations toward more favorable states. Evolutionary control extends this idea by treating treatment as a dynamic decision-making problem in which interventions can be optimized and updated as the population evolves.

One such strategy is sequential therapy, in which drugs are administered in a planned order to exploit collateral-sensitivity relationships and reduce the likelihood of resistance evolution. Optimizing such therapies can be framed as a sequential decision-making problem, where treatment choices are updated over time based on the evolving population state. Several studies have implemented this framework using Markov decision processes (MDPs) or reinforcement learning to identify optimal drug sequences that minimize resistance evolution from empirical and synthetic fitness landscapes, an invaluable area of study in cancer [41,53,87,88].

Effective steering also requires recognizing that treatment changes the selective environment itself. Fitness seascapes capture this dependence of genotype fitness on changing environmental conditions. King et al. [59,60] demonstrated that incorporating pharmacokinetic dynamics and spatial diffusion into agent-based simulations on empirical seascapes can produce highly heterogeneous evolutionary outcomes, emphasizing that control strategies must account for time- and space-dependent selection rather than relying solely on a static genotype–fitness map.

**Figure 3:**
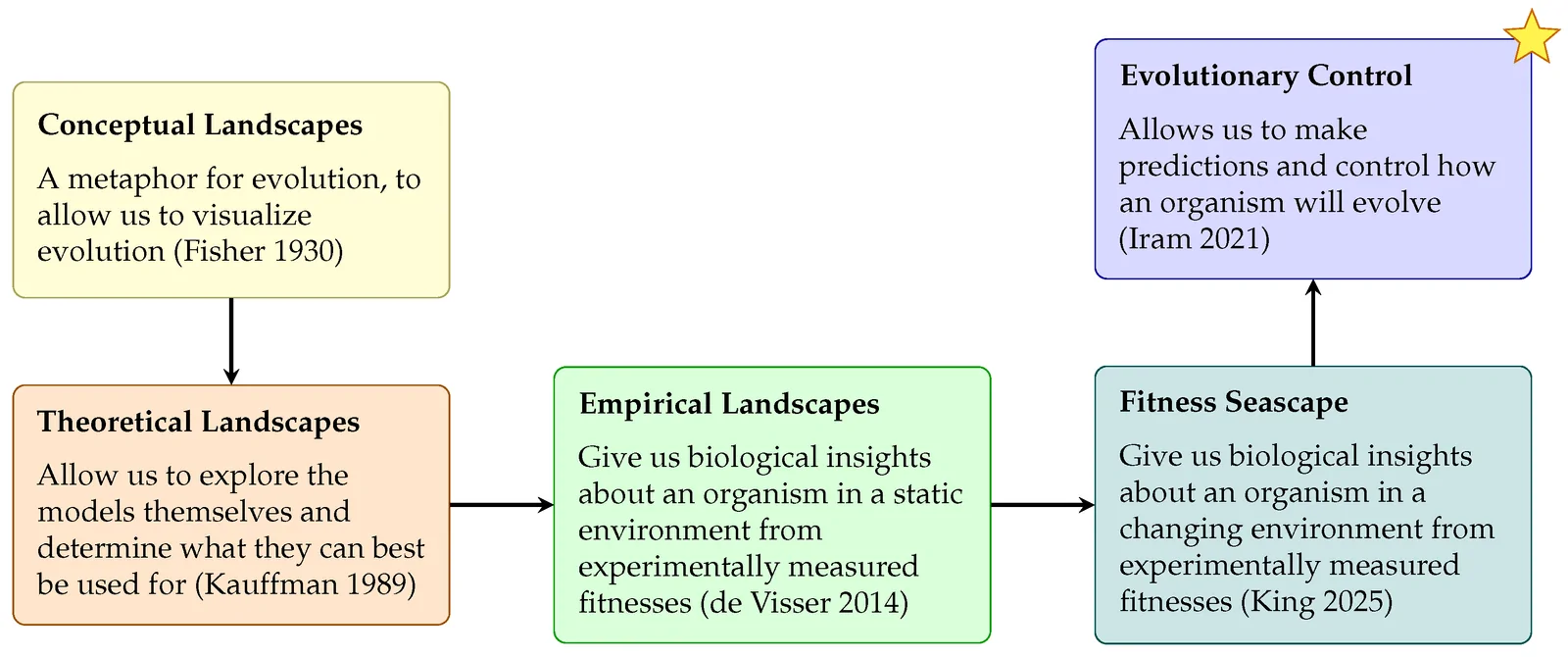
Historical expansion of the roles and applications of fitness-landscape frameworks. Fitness landscapes were initially used as conceptual representations of evolution and were subsequently developed as theoretical and empirical tools, extended to dynamically changing environments through fitness seascapes, and increasingly used to motivate evolutionary prediction, steering, and control. These categories represent overlapping roles and applications rather than mutually exclusive types of fitness landscapes [1,40,44,60,91].

Building on these predictive and empirical advances, several theoretical frameworks now treat fitness landscapes and seascapes not only as descriptive tools but also as substrates for intervention. Reinforcement learning approaches have been proposed to learn adaptive dosing policies that respond to stochastic evolutionary dynamics, aiming to suppress emergent resistant subpopulations while minimizing cumulative drug exposure [90]. In parallel, control-theoretic approaches such as counterdiabatic driving operate on explicitly time-dependent fitness seascapes, directly reshaping selection pressures to regulate evolutionary trajectories [91]. Importantly, many current demonstrations of landscape-based evolutionary steering and control remain theoretical, computational, or microbial proofs of principle; comparable applications in cancer, particularly in clinical settings, remain limited. Together, these approaches illustrate a progression from predicting likely evolutionary outcomes, to steering populations through informed treatment choices, and ultimately to controlling evolutionary trajectories through dynamically optimized interventions. This shift in the role of fitness landscapes is summarized in Figure 3. This figure shows the order in which new methods and applications have been incorporated into the field of fitness landscapes.

### 6.2. Future Directions

Looking forward, translating evolutionary control strategies from theory to practice will require richer empirical characterization of dynamic fitness landscapes. In particular, constructing large-scale empirical fitness seascapes that capture both genetic interactions and time-varying environments remains technically challenging. Progress will depend on integrating high-throughput evolution experiments, time-resolved phenotyping, pharmacokinetic modeling, and scalable inference methods capable of reconstructing dynamic genotype–fitness mappings from incomplete data. Such advances will be essential for transforming empirical fitness landscapes and seascapes into predictive and experimentally testable frameworks for steering evolution in biomedical systems. This is particularly applicable to cancer research, where the goal is to guide or prevent the development of treatment resistance in a tumor.

An additional challenge is that many theoretical results concerning evolutionary accessibility, adaptive walks, and trapping at local optima are derived under SSWM-like assumptions, in which populations are effectively monomorphic between successive fixation events. Tumors, however, often contain multiple competing subclones and substantial spatial and ecological heterogeneity, so several lineages may evolve simultaneously rather than following a single adaptive walk. Future landscape-based approaches for cancer should therefore extend beyond SSWM-like assumptions to incorporate clonal interference, population heterogeneity, and spatially structured evolution.

## 7. Discussion

In this review, we demonstrate how fitness landscapes have evolved from a simple method for visualizing the concept of evolution, to a range of formal theoretical frameworks, to empirical tools for quantifying fitness relationships and evolutionary dynamics, and more recently toward approaches for predicting and potentially steering evolution, with a focus on demonstrating their potential utility in cancer research. We have laid out the theory behind several influential fitness landscapes and explained how they have been extended over the years to incorporate new insights into biology, particularly our evolving understanding of epistasis. The wide variety of landscapes, from smooth to rugged, supports a broad range of applications and offers many theoretical insights. At present, fitness landscapes are well-positioned for a transition from purely theoretical landscapes to those incorporating experimental and clinical data.

Fitness landscapes are a broad range of models, many of which have been extensively researched theoretically, making them potentially useful frameworks for studying collateral sensitivity, adaptive therapy, and other evolutionary steering and control strategies for cancer treatment. As discussed in Section 6.1, theoretical, computational, and experimental studies provide proofs of principle that landscape structure can be exploited to predict or influence evolutionary trajectories; however, translation of these approaches to cancer treatment remains at an early stage. Another research opportunity is to use FGM to identify important phenotypes in cancer by leveraging the landscape’s dimensions and structure, generated from experimental data. Such work could help bridge the gap between theoretical or experimental demonstrations of evolutionary steering and clinically applicable strategies for limiting treatment resistance.

While the goal of this review is to demonstrate the potential of these models, particularly in the area of evolutionary steering in cancer, it should still be noted that there are many limitations and challenges when using fitness landscapes. For example, fitness itself is a concept that can be hard to define and can take many forms, from growth rate to resistance. Additionally, cancer itself is a complex illness that involves many different genes, making the scope of landscapes immense. Other challenges include that cancer is heavily influenced by its environment, a dynamic that can be hard to quantify and measure, but may make it an ideal target for fitness seascapes, and the many factors that must be considered when deciding on treatment in a clinical setting.

The hope is that this review will encourage researchers to view fitness landscapes not only as a useful framework for conceptualizing evolution, but also as a valuable method for interpreting experimental data and predicting how cancer will evolve and, ultimately, for developing strategies that steer evolutionary dynamics toward more favorable outcomes. Translating such approaches into patient-specific evolutionary control, however, remains an important future goal.

## 8. Conclusions

As demonstrated in this review, fitness landscapes have become valuable frameworks for studying evolution across a wide range of biological systems. In oncology, they can improve our understanding of cancer evolution and the development of treatment resistance by linking evolutionary dynamics to genetic interactions and changing environments. Although clinical applications of evolutionary control remain limited, continued development of empirical and dynamic landscapes may help translate these ideas into clinically useful strategies. Ultimately, fitness landscapes may provide a framework not only for understanding cancer evolution, but also for predicting and ultimately controlling it.

## Figures and Tables

**Table 1:** Representative empirical fitness-landscape studies were selected to illustrate the diversity of biological systems (microbial, protein, viral, and cancer systems), experimental approaches, landscape contexts, fitness proxies, and key empirical findings, rather than to provide a systematic survey of the literature.

Study	Biological System	Genotypes	Landscape Context(s)	Fitness Proxy ^†^	Key Insight
Microbial systems					
Hietpas et al. 2012 [65]	Yeast	Saturation single-codon mutants	Single genetic background	Selection coefficient	Established the EMPIRIC platform for systematic measurement of point-mutation fitness effects
Schenk et al. 2013 [66]	*E. coli* (TEM-1 β-lactamase)	16 genotypes each	2 genetic backgrounds	Cefotaxime resistance	Demonstrated sign epistasis constrains accessible adaptive trajectories
Mira et al. 2015 [67]	*E. coli* (TEM-50 β-lactamase)	16 genotypes	15 antibiotic environments	Growth rate	Demonstrated genotype-by-environment interactions reshape adaptive landscapes
Ogbunugafor et al. 2016 [61]	*Plasmodium falciparum* DHFR	16 genotypes	2 environmental conditions	Growth rate	Demonstrated environment-dependent fitness landscapes across drug conditions
Bajic et al. 2018 [68]	*E. coli*	∼10^7^ mutants	Single metabolic environment	Simulated growth rate (dFBA)	Demonstrated eco-evolutionary feedback reshapes fitness landscapes through metabolic niche construction
Johnson et al. 2019 [70]	Yeast	163 backgrounds with 91 mutations	Multiple genetic backgrounds	Competitive growth fitness	Demonstrated adaptation reduces mutational robustness
Iwasawa et al. 2022 [69]	*E. coli*	44 evolved lineages	8 antibiotics	Drug resistance (IC50)	Constructed phenotype-based fitness landscapes linking genotype and drug resistance
Diaz-Colunga et al. 2023 [71]	*Plasmodium falciparum*	15 genotypes	Single experimental environment	Relative growth rate/fitness	Demonstrated that environmental variation reshapes global epistasis
Guerrero et al. 2024 [72]	β-lactamase resistance system	16 alleles	7 environmental conditions	Relative growth rate	Demonstrated environmental epistasis as a major determinant of resistance evolution
Protein-level					
Weinreich et al. 2006 [39]	*E. coli* (TEM β-lactamase)	32 genotypes	Single antibiotic environment	Cefotaxime MIC	Demonstrated sign epistasis restricts accessible paths to antibiotic resistance
Meini et al. 2015 [73]	Metallo-β-lactamase BcII	16 genotypes	Single antibiotic environment	Cephalexin MIC	Constructed an empirical protein fitness landscape
Wu et al. 2016 [74]	GB1 protein	160,000 variants	Single experimental condition	Relative protein fitness	Generated higher-dimensional empirical protein landscapes using deep mutational scanning
Chen et al. 2023 [75]	Multiple proteins	Multiple DMS variant sets	36 protein DMS datasets	Assay-dependent DMS fitness	Demonstrated multi-protein learning improves variant-effect prediction across proteins
Viral systems					
Sanjuán et al. 2004 [76]	Vesicular stomatitis virus	91 single-nucleotide substitution mutants	2 host environments	Relative growth rate	Demonstrated high mutational fragility in RNA virus fitness landscapes
Lauring et al. 2011 [77]	Poliovirus	48 variants	Single experimental environment	Relative competitive fitness	Characterized mutational robustness in the poliovirus fitness landscape
Acevedo et al. 2014 [78]	Poliovirus	∼8970 single-nucleotide variants	Single experimental environment	Allele frequency dynamics	Demonstrated RNA virus populations form a mutational cloud defined by a distribution of fitness effects
Muñoz-Moreno et al. 2019 [79]	Influenza A virus	56 NS1 variants	3 experimental environments	Barcode abundance	Constructed phenotype-level viral fitness landscapes across environments
Cancer					
Rogers et al. 2018 [81]	KRAS-driven lung adenocarcinoma	31 mutations	3 genetic backgrounds	Tumor growth/size	Demonstrated genetic background reshapes the cancer fitness landscape
Salehi et al. 2021 [82]	Breast cancer	6–11 clones per landscape	Untreated and cisplatin-treated contexts	Clonal abundance	Reconstructed clonal fitness landscapes from evolving cancer populations
Yousefi et al. 2023 [83]	Lung adenocarcinoma	8 genotypes	Multiple genetic backgrounds	Relative tumor growth rate	Constructed an empirical fitness landscape for lung adenocarcinoma evolution
Blair et al. 2023 [45]	Lung adenocarcinoma	∼112 mutations	4 oncogenic backgrounds	Relative tumor size	Demonstrated context-dependent epistasis between oncogenes and tumor suppressors

^†^ Fitness proxy denotes the experimentally measured or inferred quantity used to operationalize fitness in each study. These proxies may capture distinct dimensions of evolutionary fitness and should not be interpreted as directly equivalent.

## Data Availability

No new data were generated or analyzed in this study. Data sharing is not applicable to this article.

## References

[1] Fisher R. (1930). The Genetical Theory of Natural Selection: A Complete Variorum Edition.

[2] Wright S. (1932). The Roles of Mutation, Inbreeding, crossbreeding and Selection in Evolution. Proc. XI Int. Congr. Genet..

[3] Waddington C.H. (1942). Canalization of development and the inheritance of acquired Characters. Nature.

[4] Fitch W.M., Ayala F.J. (1995). Tempo and Mode in Evolution: Genetics and Paleontology 50 Years After Simpson.

[5] Orr H.A. (1998). The population genetics of adaptation: The distribution of factors fixed during adaptive evolution. Evolution.

[6] Orr H.A. (2000). Adaptation and the cost of complexity. Evolution.

[7] Orr H.A. (2005). The genetic theory of adaptation: A brief history. Nat. Rev. Genet..

[8] Kauffman S., Levin S. (1987). Towards a general theory of adaptive walks on rugged landscapes. J. Theor. Biol..

[9] Aita T., Uchiyama H., Inaoka T., Nakajima M., Kokubo T., Husimi Y. (2000). Analysis of a local fitness landscape with a model of the rough Mt. Fuji-type landscape: Application to prolyl endopeptidase and thermolysin. Biopolymers.

[10] Hirst J.D. (1999). The evolutionary landscape of functional model proteins. Protein Eng. Des. Sel..

[11] Weinreich D.M., Watson R.A., Chao L. (2005). PERSPECTIVE: SIGN EPISTASIS AND GENETIC COSTRAINT ON EVOLUTIONARY TRAJECTORIES. Evolution.

[12] Mustonen V., Lässig M. (2009). From fitness landscapes to seascapes: Non-equilibrium dynamics of selection and adaptation. Trends Genet..

[13] Kingman J.F.C. (1978). A Simple Model for the Balance between Selection and Mutation. J. Appl. Probab..

[14] MacColl A.D.C. (2011). The ecological causes of evolution. Trends Ecol. Evol..

[15] Bateson W., Seward A.C. (1909). Heredity and variation in modern lights. Darwin and Modern Science.

[16] Phillips P.C. (2008). Epistasis—The essential role of gene interactions in the structure and evolution of genetic systems. Nat. Rev. Genet..

[17] Bank C. (2022). Epistasis and Adaptation on Fitness Landscapes. Annu. Rev. Ecol. Evol. Syst..

[18] Matlak D., Szczurek E. (2017). Epistasis in genomic and survival data of cancer patients. PLoS Comput. Biol..

[19] van de Haar J., Canisius S., Yu M.K., Voest E.E., Wessels L.F., Ideker T. (2019). Identifying Epistasis in Cancer Genomes: A Delicate Affair. Cell.

[20] Wang X., Fu A.Q., McNerney M.E., White K.P. (2014). Widespread genetic epistasis among cancer genes. Nat. Commun..

[21] Gillespie J.H. (1984). Molecular Evolution Over the Mutational Landscape. Evolution.

[22] Poelwijk F.J., Tănase-Nicola S., Kiviet D.J., Tans S.J. (2011). Reciprocal sign epistasis is a necessary condition for multi-peaked fitness landscapes. J. Theor. Biol..

[23] Weinreich D.M., Lan Y., Jaffe J., Heckendorn R.B. (2018). The Influence of Higher-Order Epistasis on Biological Fitness Landscape Topography. J. Stat. Phys..

[24] Diaz-Colunga J., Skwara A., Gowda K., Diaz-Uriarte R., Tikhonov M., Bajic D., Sanchez A. (2023). Global epistasis on fitness landscapes. Philos. Trans. R. Soc. B Biol. Sci..

[25] Krug J., Baake E., Baake E.W.A. (2021). Accessibility percolation in random fitness landscapes. Probabilistic Structures in Evolution.

[26] Carter A.J., Hermisson J., Hansen T.F. (2005). The role of epistatic gene interactions in the response to selection and the evolution of evolvability. Theor. Popul. Biol..

[27] Draghi J.A., Plotkin J.B. (2013). Selection biases the prevalence and type of epistasis along adaptive trajectories. Evolution.

[28] ORR H.A. (1999). The evolutionary genetics of adaptation: A simulation study. Genet. Res..

[29] Martin G., Lenormand T. (2006). A general multivariate extension of Fisher’s geometrical model and the distribution of mutation fitness effects across species. Evol. Int. J. Org. Evol..

[30] Perfeito L., Sousa A., Bataillon T., Gordo I. (2014). RATES OF FITNESS DECLINE AND REBOUND SUGGEST PERVASIVE EPISTASIS. Evolution.

[31] Keagy J., Lettieri L., Boughman J.W. (2016). Male competition fitness landscapes predict both forward and reverse speciation. Ecol. Lett..

[32] Blanquart F., Bataillon T. (2016). Epistasis and the Structure of Fitness Landscapes: Are Experimental Fitness Landscapes Compatible with Fisher’s Geometric Model?. Genetics.

[33] Martin G., Elena S.F., Lenormand T. (2007). Distributions of epistasis in microbes fit predictions from a fitness landscape model. Nat. Genet..

[34] Harmand N., Gallet R., Jabbour-Zahab R., Martin G., Lenormand T. (2017). Fisher’s geometrical model and the mutational patterns of antibiotic resistance across dose gradients. Evolution.

[35] Weinreich D.M., Knies J.L. (2013). FISHER’S GEOMETRIC MODEL OF ADAPTATION MEETS THE FUNCTIONAL SYNTHESIS: DATA ON PAIRWISE EPISTASIS FOR FITNESS YIELDS INSIGHTS INTO THE SHAPE AND SIZE OF PHENOTYPE SPACE. Evolution.

[36] Razo-Mejia M., Mani M., Petrov D.A. (2026). Learning the shape of evolutionary landscapes: Geometric deep learning reveals hidden structure in phenotype-to-fitness maps. PRX Life.

[37] Wright S. (1931). Evolution in Mendelian Populations. Genetics.

[38] Aita T., Husimi Y. (1998). Adaptive Walks by the Fittest among Finite Random Mutants on a Mt. Fuji-type Fitness Landscape. J. Theor. Biol..

[39] Weinreich D.M., Delaney N.F., DePristo M.A., Hartl D.L. (2006). Darwinian evolution can follow only very few mutational paths to fitter proteins. Science.

[40] de Visser J.A.G.M., Krug J. (2014). Empirical fitness landscapes and the predictability of evolution. Nat. Rev. Genet..

[41] Weaver D.T., King E.S., Maltas J., Scott J.G. (2024). Reinforcement learning informs optimal treatment strategies to limit antibiotic resistance. Proc. Natl. Acad. Sci. USA.

[42] Diaz-Uriarte R. (2017). Cancer progression models and fitness landscapes: A many-to-many relationship. Bioinformatics.

[43] Diaz-Uriarte R., Vasallo C. (2019). Every which way? On predicting tumor evolution using cancer progression models. PLoS Comput. Biol..

[44] Kauffman S.A., Weinberger E.D. (1989). The NK model of rugged fitness landscapes and its application to maturation of the immune response. J. Theor. Biol..

[45] Blair L.M., Juan J.M., Sebastian L., Tran V.B., Nie W., Wall G.D., Gerceker M., Lai I.K., Apilado E.A., Grenot G. (2023). Oncogenic context shapes the fitness landscape of tumor suppression. Nat. Commun..

[46] Weinberger E.D. (1991). Local Properties of Kauffman’s N-K Model: A Tunably Rugged Energy Landscape. Phys. Rev. A.

[47] Hordijk W., Kauffman S.A. (2005). Correlation analysis of coupled fitness landscapes. Complexity.

[48] Hwang S., Schmiegelt B., Ferretti L., Krug J. (2018). Universality classes of interaction structures for NK fitness landscapes. J. Stat. Phys..

[49] Song S., Zhang J. (2021). Unbiased inference of the fitness landscape ruggedness from imprecise fitness estimates. Evolution.

[50] Neidhart J., Szendro I.G., Krug J. (2014). Adaptation in Tunably Rugged Fitness Landscapes: The Rough Mount Fuji Model. Genetics.

[51] Maltas J., McNally D.M., Wood K.B. (2021). Evolution in alternating environments with tunable interlandscape correlations. Evolution.

[52] Chen P., Krishnan N., Stacy A., Weaver D.T., Barker-Clarke R., Hinczewski M., Maltas J., Scott J.G. (2025). Inverted topographies in sequential fitness landscapes enable evolutionary control. bioRxiv.

[53] Chen P., Pachter J.A., Scott J.G., Hinczewski M. (2026). Stochastic Evolutionary Control in Heterogeneous Populations. bioRxiv.

[54] Merrell D.J. (1994). The Adaptive Seascape: The Mechanism of Evolution.

[55] Trubenová B., Krejca M.S., Lehre P.K., Kötzing T. (2019). Surfing on the seascape: Adaptation in a changing environment. Evolution.

[56] Das S.G., Direito S.O., Waclaw B., Allen R.J., Krug J. (2020). Predictable properties of fitness landscapes induced by adaptational tradeoffs. eLife.

[57] Lindsey H.A., Gallie J., Taylor S., Kerr B. (2013). Evolutionary rescue from extinction is contingent on a lower rate of environmental change. Nature.

[58] Ottino-Löffler B., Kardar M. (2020). Population extinction on a random fitness seascape. Phys. Rev. E.

[59] King E.S., Tadele D.S., Pierce B., Hinczewski M., Scott J.G. (2024). Diverse mutant selection windows shape spatial heterogeneity in evolving populations. PLoS Comput. Biol..

[60] King E.S., Stacy A.E., Weaver D.T., Maltas J., Barker-Clarke R., Dolson E., Scott J.G. (2025). Fitness seascapes are necessary for realistic modeling of the evolutionary response to drug therapy. Sci. Adv..

[61] Ogbunugafor C.B., Wylie C.S., Diakite I., Weinreich D.M., Hartl D.L. (2016). Adaptive Landscape by Environment Interactions Dictate Evolutionary Dynamics in Models of Drug Resistance. PLoS Comput. Biol..

[62] Gjini E., Wood K.B. (2021). Price equation captures the role of drug interactions and collateral effects in the evolution of multidrug resistance. eLife.

[63] Das S.G., Krug J., Mungan M. (2022). Driven Disordered Systems Approach to Biological Evolution in Changing Environments. Phys. Rev. X.

[64] Das S.G., Mungan M., Krug J. (2025). Epistasis-mediated compensatory evolution in a fitness landscape with adaptational tradeoffs. Proc. Natl. Acad. Sci. USA.

[65] Hietpas R., Roscoe B., Jiang L., Bolon D.N.A. (2012). Fitness analyses of all possible point mutations for regions of genes in yeast. Nat. Protoc..

[66] Schenk M.F., Szendro I.G., Salverda M.L.M., Krug J., de Visser J.A.G.M. (2013). Patterns of epistasis between beneficial mutations in an antibiotic resistance gene. Mol. Biol. Evol..

[67] Mira P.M., Meza J.C., Nandipati A., Barlow M. (2015). Adaptive Landscapes of Resistance Genes Change as Antibiotic Concentrations Change. Mol. Biol. Evol..

[68] Bajić D., Vila J.C.C., Blount Z.D., Sánchez A. (2018). On the deformability of an empirical fitness landscape by microbial evolution. Proc. Natl. Acad. Sci. USA.

[69] Iwasawa J., Maeda T., Shibai A., Kotani H., Kawada M., Furusawa C. (2022). Analysis of the evolution of resistance to multiple antibiotics enables prediction of the Escherichia coli phenotype-based fitness landscape. PLoS Biol..

[70] Johnson M.S., Martsul A., Kryazhimskiy S., Desai M.M. (2019). Higher-fitness yeast genotypes are less robust to deleterious mutations. Science.

[71] Diaz-Colunga J., Sanchez A., Ogbunugafor C.B. (2023). Environmental modulation of global epistasis in a drug resistance fitness landscape. Nat. Commun..

[72] Guerrero R.F., Dorji T., Harris R.M., Shoulders M.D., Ogbunugafor C.B. (2024). Evolutionary druggability for low-dimensional fitness landscapes toward new metrics for antimicrobial applications. eLife.

[73] Meini M.R., Tomatis P.E., Weinreich D.M., Vila A.J. (2015). Quantitative description of a protein fitness landscape based on molecular features. Mol. Biol. Evol..

[74] Wu N.C., Dai L., Olson C.A., Lloyd-Smith J.O., Sun R. (2016). Adaptation in protein fitness landscapes is facilitated by indirect paths. eLife.

[75] Chen L., Zhang Z., Li Z., Li R., Huo R., Chen L., Wang D., Luo X., Chen K., Liao C. (2023). Learning protein fitness landscapes with deep mutational scanning data from multiple sources. Cell Syst..

[76] Sanjuán R., Moya A., Elena S.F. (2004). The distribution of fitness effects caused by single-nucleotide substitutions in an RNA virus. Proc. Natl. Acad. Sci. USA.

[77] Lauring A.S., Andino R. (2011). Exploring the fitness landscape of an RNA virus by using a universal barcode microarray. J. Virol..

[78] Acevedo A., Brodsky L., Andino R. (2014). Mutational and fitness landscapes of an RNA virus revealed through population sequencing. Nature.

[79] Muñoz-Moreno R., Martínez-Romero C., Blanco-Melo D., Forst C.V., Nachbagauer R., Benitez A.A., Mena I., Aslam S., Balasubramaniam V., Lee I. (2019). Viral fitness landscapes in diverse host species reveal multiple evolutionary lines for the NS1 gene of influenza A viruses. Cell Rep..

[80] Mohanty V., Shakhnovich E.I. (2026). Biophysical fitness landscape design traps viral evolution. Proc. Natl. Acad. Sci. USA.

[81] Rogers Z.N., McFarland C.D., Winters I.P., Seoane J.A., Brady J.J., Yoon S., Curtis C., Petrov D.A., Winslow M.M. (2018). Mapping the in vivo fitness landscape of lung adenocarcinoma tumor suppression in mice. Nat. Genet..

[82] Salehi S., Kabeer F., Ceglia N., Andronescu M., Williams M.J., Campbell K.R., Masud T., Wang B., Biele J., Brimhall J. (2021). Clonal fitness inferred from time-series modelling of single-cell cancer genomes. Nature.

[83] Yousefi M., Andrejka L., Szamecz M., Winslow M.M., Petrov D.A., Boross G. (2023). Fully accessible fitness landscape of oncogene-negative lung adenocarcinoma. Proc. Natl. Acad. Sci. USA.

[84] Cannataro V.L., Gaffney S.G., Townsend J.P. (2018). Effect Sizes of Somatic Mutations in Cancer. J. Natl. Cancer Inst..

[85] Cross W., Kovac M., Mustonen V., Temko D., Davis H., Baker A.M., Biswas S., Arnold R., Chegwidden L., Gatenbee C. (2018). The evolutionary landscape of colorectal tumorigenesis. Nat. Ecol. Evol..

[86] Barbosa C., Trebosc V., Kemmer C., Rosenstiel P., Beardmore R., Schulenburg H., Jansen G. (2017). Alternative Evolutionary Paths to Bacterial Antibiotic Resistance Cause Distinct Collateral Effects. Mol. Biol. Evol..

[87] Maltas J., Wood K.B. (2019). Pervasive and diverse collateral sensitivity profiles inform optimal strategies to limit antibiotic resistance. PLoS Biol..

[88] Maltas J., Huynh A., Wood K.B. (2025). Dynamic collateral sensitivity profiles highlight opportunities and challenges for optimizing antibiotic treatments. PLoS Biol..

[89] Nichol D., Rutter J., Bryant C., Hujer A.M., Lek S., Adams M.D., Jeavons P., Anderson A.R.A., Bonomo R.A., Scott J.G. (2019). Antibiotic collateral sensitivity is contingent on the repeatability of evolution. Nat. Commun..

[90] Engelhardt D. (2020). Dynamic Control of Stochastic Evolution: A Deep Reinforcement Learning Approach to Adaptively Targeting Emergent Drug Resistance. J. Mach. Learn. Res..

[91] Iram S., Dolson E., Chiel J., Pelesko J., Krishnan N., Güngör Ö., Kuznets-Speck B., Deffner S., Ilker E., Scott J.G. (2021). Controlling the speed and trajectory of evolution with counterdiabatic driving. Nat. Phys..

